# Bioactive multi-protein adsorption enables targeted mast cell nanotherapy

**DOI:** 10.21203/rs.3.rs-2468299/v1

**Published:** 2023-01-26

**Authors:** Fanfan Du, Clayton H. Rische, Yang Li, Michael P. Vincent, Rebecca A. Krier-Burris, Yuan Qian, Simseok A. Yuk, Sultan Almunif, Bruce S. Bochner, Baofu Qiao, Evan A. Scott

**Affiliations:** 1 Department of Biomedical Engineering, Northwestern University, Evanston, IL 60208, USA.; 2 Department of Medicine, Division of Allergy and Immunology, Northwestern University Feinberg School of Medicine, Chicago, IL 60611, USA.; 3 Department of Chemical Engineering, Northwestern University, Evanston, Illinois 60201, USA.; 4 Department of Materials Science and Engineering, Northwestern University, Evanston, IL 60208, USA.; 5 Department of Natural Sciences, Baruch College, City University of New York, New York, NY 10010, USA.; 6 Simpson Querrey Institute, Northwestern University, Chicago, IL 60611, USA.; 7 Chemistry of Life Processes Institute, Northwestern University, Evanston, IL 60208, USA.; 8 Interdisciplinary Biological Sciences Program, Northwestern University, Evanston, IL 60208, USA.; 9 Robert H. Lurie Comprehensive Cancer Center, Northwestern University, Chicago, IL 60611, USA.

Proteins readily and often irreversibly adsorb to nanomaterial surfaces, resulting in denaturation and loss of bioactivity^1,2^. Controlling this process to preserve protein structure and function has remained an elusive goal that would enhance the fabrication and biocompatibility of protein-based bioactive nanomaterials^3–7^. Here, we demonstrate that poly(propylene sulfone) (PPSU)^8^ nanoparticles support the controlled formation of multi-component enzyme and antibody coatings while maintaining their bioactivity. Simulations indicate that hydrophobic patches^9^ on protein surfaces induce site-specific dipole relaxation on PPSU surfaces to noncovalently anchor proteins without disrupting hydrogen bonding or protein structure. As proof-of-concept, a nanotherapy for enhanced antibody-based targeting of mast cells and inhibition of anaphylaxis^3,4^ is demonstrated in a humanized mouse model. The ratio of co-adsorbed anti-Siglec-6^10,11^ and anti-FcεRIα antibodies is systematically optimized to effectively inhibit mast cell activation and degranulation. Protein immobilization on PPSU surfaces therefore provides a simple and rapid platform for the development of targeted nanomedicines.

Nanomaterials that preserve the bioactivity of anchored proteins hold promise for a wide range of applications as nanozymes, diagnostic sensors, and targeted delivery systems^5–7^. Although various synthetic platforms have been developed, unmodified nanoparticles (NPs) remain challenging in interfacing with a wide range of proteins without affecting protein folding or function^7,12^. For example, NPs with hydrophobic surfaces tend to irreversibly adsorb protein adlayers, leading to denaturation and associated loss of protein bioactivity^1,2^. It is therefore mandatory to involve hydrophilic “anti-fouling” polymers, such as poly(ethylene glycol) (PEG)^13^ and zwitterionic moieties^14,15^, within NP surfaces to prevent this non-specific adsorption.

Given their highly hydrophilic surfaces after modifications, most NPs require the tedious process of chemical coupling to reliably interface with proteins to harness their bioactivity^3–7^. This method has simultaneously enabled the surface display of targeting antibodies but also limited the control to typically a single antibody type with minimal control over the surface density. Protein adsorption thus presents a comparatively facile noncovalent approach to rapidly engineer bioactive NP surfaces with multiple antibodies and proteins. However, application of this approach is hampered by the continued desorption or loss of activity of the protein layer^16–18^. Of note, controlling the composition and stability of anchored proteins, particularly for multiple antibodies, has remained challenging even when employing chemical coupling strategies, which have conjugation yields dependent on the reactivity of the NP surface and accessibility of binding sites within proteins.

We report on PPSU NPs that irreversibly adsorb nonspecific proteins without compromising protein function. This ready-to-use protein immobilization platform is effective for both single and multi-component antibody and enzyme coatings with well-controlled composition. To further demonstrate this design freedom, we engineer PPSU NPs into the first antibody-based nanomedicine for desensitizing mast cells (MCs) to allergen. The nanotherapy requires the controlled co-adsorption of two separate antibodies at an optimized ratio to tune the simultaneous signaling of Siglec-6 and FcεRIα receptors in close proximity on MC surfaces. This therapeutic strategy administers allergen immunotherapy without triggering anaphylaxis in a humanized mouse model.

Hollow PPSU NPs (Fig. 1a) were fabricated at scale (Supplementary Fig. 1) according to our previous report^8^. As prepared, the NPs are negatively charged on their surfaces with a zeta potential of ~ −39 mV in water. We ascribed this phenomenon to spontaneous orientation of propylene sulfone (PS, Fig. 1b) at the interface between water and PPSU due to inherent amphiphilicity within the repeating unit. To explore this postulate, explicit solvent all-atom molecular dynamics (AAMD) simulations of PPSU in water were performed to construct a hollow aggregate that mimics the experimentally observed PPSU NPs. Hollow NPs formed in both the 125-chain and 600-chain simulation systems (Fig. 1c) without severe deformation or collapse of shell structures (Supplementary Fig. 2). By calculation of the Coulombic (polar) and Lennard-Jones (nonpolar) interactions between PPSU and water (Supplementary Fig. 3), we confirmed increased surface hydrophilicity for simulated NPs when compared to a single PPSU chain (Fig. 1c). Thus, we concluded spontaneous orientation of PSs at the water-PPSU interface.

The simulations showed that the PPSU NPs exhibit comparable or even higher surface hydrophilicity compared to that of PEG. However, we were surprised to observe the rapid adsorption of nonspecific proteins by PPSU NPs in phosphate buffered saline (PBS) (Supplementary Fig. 4). Protein surfaces are chemically diverse with characteristic distributions of surface accessible hydrophobic patches (Fig. 1d)^9^. Because the hydrophilic state of PPSU surfaces is induced by the aqueous solvent molecules, we inferred that the dominant orientations of interfacial PSs could also be directed by the hydration of adjacent patches experienced at protein interfaces. That is, a hydrophobic protein patch is likely to flip the interfacial PSs while a hydrated hydrophilic patch would preserve the hydrophilic state of PPSU surfaces (Fig. 1e). Such local hydrophilic/hydrophobic switching events thermodynamically favor the screening of electrostatic repulsion among sulfone groups, leading to small site-to-site heterogeneous NP surfaces at PPSU-protein interfaces.

To investigate the mechanism of protein adsorption, the AAMD simulation of the 600-chain PPSU NP was proceeded by adding 6 molecules (the limit set by space constraints) of trypsin in the aqueous system. The last simulation snapshot is shown in Fig. 2a and Supplementary Movie 1. All six trypsin molecules were adsorbed by the PPSU NP. Calculations of trypsin-NP interactions explicitly supported that the hydrophobic interactions were a major determinant of the adsorption process (Fig. 2b). The 3D structure of adsorbed trypsin was preserved at the PPSU surface, supported by the small root-mean-square deviation of approximately 1.5 Å for each trypsin molecule during the simulation (Supplementary Fig. 5)^19^. This is in contrast with traditional hydrophobic surfaces where proteins were often found to spread and unfold upon hydrophobic binding (Fig. 1e)^2^. Furthermore, the 6 trypsin molecules were randomly oriented after anchoring (Fig. 2c, Supplementary Fig. 6). The non-specific orientation of trypsin allowed the exposure of their active sites for 5 out of the 6 molecules, demonstrating retention of enzymatic activity even when shielding by further corona formation was taken into account^16^. Dipole relaxation of the PPSU backbone at all 6 PPSU-trypsin interfaces was confirmed via calculating the surface percentage of sulfone groups, revealing significantly enhanced surface hydrophobicity triggered by trypsin adsorption (Fig. 2d, Supplementary Fig. 7). Simulations also showed that water lubricated the PPSU-trypsin contact regime via PPSU-water-trypsin H-bonds (Fig. 2a, inset). Furthermore, the hydration of trypsin, described by the amounts of trypsin-water H-bonds and water neighbors, remained unchanged despite hydrophobic adsorption (Fig. 2e). This retention of protein hydration is an appealing feature for hydrophilic surfaces and essential for preserving the bioactivity of proteins^19^. Taken together, the simulation results suggested that restructuring of the PPSU surface, rather than denaturing the adsorbed trypsin, led to controlled interfacial hydrophobic interactions for preserving protein structure. Of note, similar results were reported by simulating the PPSU NP in a bovine serum albumin (BSA) solution (Supplementary Fig. 8).

Having understood the distinct protein affinity of PPSU surfaces, we established a facile and versatile process for coating PPSU NPs with protein adlayers (Fig. 3a). In brief, PPSU NPs were incubated with excess proteins in PBS for 5 min at room temperature, followed by thorough washing to remove dynamically adsorbed proteins as well as residual proteins in solution. Saturation of adsorbed proteins on the NPs avoided aggregation in PBS (Supplementary Fig. 9). The obtained protein-coated NPs showed improved colloidal stability, with their zeta potential dependent on the adsorbed proteins (Fig. 3b). Using BSA as a model protein, small-angle X-ray scattering (SAXS) measurements revealed that the shell thickness of NPs increased from 5.3 nm to 7.1 nm after protein coating (Fig. 3c). The BSA-coated NPs were imaged by transmission electron microscopy (TEM) and cryogenic scanning TEM (cryo-STEM), showing consistent sizes and morphologies with that of the pristine PPSU NPs (Supplementary Fig. 10). To probe the displacement of adsorbed proteins by competitive serum proteins, we coated PPSU NPs with fluorescein isothiocyanate-tagged BSA (FITC-BSA) and incubated the complex of FITC-BSA@NP in pooled human plasma for 48 h at 37 °C. FITC-BSA fluorescence was retained on pelleted NPs but was undetectable in the supernatant, confirming the irreversibility of the protein adsorption process (Supplementary Fig. 11).

We explored the effect of irreversible adsorption on protein bioactivity using various adsorbed proteins as models. Trypsin and green fluorescent protein (GFP) were coated onto PPSU NPs to form complexes of trypsin@NP and GFP@NP, respectively. The stable surface presentation of adsorbed trypsin was demonstrated by matrix assisted laser desorption ionization time-of-flight (MALDI-TOF) mass spectrometry (Fig. 3d). To assess the enzyme activity of trypsin@NP, we quantified the significant increase in fluorescence resulting from the proteolytic cleavage of quenched FITC-BSA solutions into FITC-labeled peptides (Supplementary Fig. 12). This assay confirmed the ability of trypsin@NP to cleave BSA (Fig. 3e). We previously reported that GFP had no detectable fluorescence after encapsulation within PPSU NPs, likely due to exposure to DMSO during NP formation^8^. In contrast, the structure-dependent fluorescence of GFP was readily detectable for GFP@NP (Fig. 3f), verifying surface adsorption to be a reliable and facile process for loading proteins without exposure to denaturing organic solvents.

We next assessed the retention of antibody binding affinity, using low mass ratios of antibodies to achieve near 100% adsorption efficiency. Given the incomplete surface coverage of the antibody-adsorbed NPs, BSA as a blocking agent, was subsequently coated to avoid non-specific interactions with unoccupied regions on the NP surface. This two-step pre-adsorption protocol allows us to incorporate various and even multiple antibodies into protein adlayers with well-controlled composition (Supplementary Fig. 13). Staining anti-CD4/BSA@NP with secondary gold-coupled antibodies verified the surface presence as well as immunological recognition by the pre-adsorbed primary antibodies (Fig. 3g, Supplementary Fig. 14). Using CD3 as a cellular target, anti-CD3/BSA@NP significantly increased uptake into T cells compared to controls (Fig. 3h).

MCs are tissue-based granulocytes involved in allergic and other responses and are historically difficult to selectively target with therapeutics^20–22^. Perhaps the most significant barrier to developing selective therapies for MCs is the difficulty identifying specific surface targets to mediate inhibitory signaling upon FcεRIα activation following allergen binding. Siglec-6 has been identified as such a target^10,11^, but optimizing inhibitory Siglec-6 signaling during FcεRIα engagement has not been achieved. We hypothesized that co-engagement of Siglec-6 with pre-adsorbed antibodies on PPSU NPs could be optimized to inhibit MC secretion for the prevention of anaphylaxis. To test this hypothesis, PPSU-based nanomedicines that consist of co-adsorbed anti-Siglec-6 and anti-FcεRIα antibodies were prepared with a controlled surface density of anti-Siglec-6 antibodies (Fig. 4a). We confirmed the bioactivity of pre-adsorbed anti-FcεRIα antibodies to bind and activate primary human skin MCs *in vitro* (Supplementary Fig. 15). Importantly, inhibited secretion was observed when the nanomedicines were administered (Fig. 4b–e, Supplementary Fig. 16). This result suggested the ability of co-localized engagement to inhibit FcεRI-mediated activation of primary human mast skin cells (Fig. 4c).

MC degranulation cell surface markers (CD107a and CD63) were quantified via flow cytometry to assess the extent of mast cell activation. The experiments showed that lower densities of anti-Siglec-6 on NP surfaces resulted in greater receptor engagement and inhibition (Fig. 4d). The treatment was further evaluated and compared to a variety of controls, including a mixture of free form anti-FcεRIα with anti-Siglec-6/BSA@NP to assess the ability of a nanotherapy to inhibit pre- or co-activated MCs. The data indicated that localized co-engagement of both Siglec-6 with FcεRIα is necessary to achieve a significant reduction in degranulation (>60%; p < 0.001), which was compared to control formulations where anti-FcεRIα was delivered without being co-adsorbed or without anti-Siglec-6 (Fig. 4e). Notably, these *in vitro* data demonstrated the need for both antibodies to be present on the NPs simultaneously.

For *in vivo* validation, we tested the optimized nanomedicine formulation in a humanized MC mouse model by intravenous injection (Supplementary Fig. 17). Anaphylactic reactions were measured by monitoring changes in body temperature using a digital rectal thermometer and a clinical scoring system (Fig. 4f)^23^. Mice that experienced anaphylaxis had significantly greater drops in body temperature and increased clinical scores (ΔT > 5°C, clinical scores > 2, p < 0.001)). Successful inhibition of anaphylaxis was manifested by responses that were statistically indistinguishable to the PBS negative control (ΔT ≤1°C, clinical score <0.5). Mice treated with the nanomedicine displayed near-complete inhibition of anaphylaxis, whereas other formulations which did not have antibodies co-adsorbed or were freely solubilized did not display any significant inhibition of anaphylaxis.

Using a combination of *in silico*, *in vitro* and *in vivo* methods and a simple mixing protocol, we demonstrate controlled, irreversible adsorption of multiple proteins simultaneously to PPSU NP surfaces while preserving protein function. Highly dynamic PPSU surfaces were capable of site-to-site hydrophilic/hydrophobic switching in the presence of adsorbing protein, which allowed facile binding of bioactive enzyme and antibody combinations. This design freedom provided the flexibility to optimize a novel nanotherapy for anaphylaxis, achieving the first targeted inhibition of FcεRI receptor activation on human mast cells via Siglec-6 signaling. Our work validates PPSU as a robust platform for the facile engineering of bioactive NPs and allows customization of the complex and combinatorial capabilities of biologics in the treatment of disease.

## Methods

### Preparation of PPSU NPs.

PPSU was prepared in our previous report^8^. Self-assembly of PPSU was performed via stepwise hydration using the confined impingement jets mixer. Typically, 15 mL of PPSU solution (25 mg/mL in DMSO) and 15 mL of water were impinged against one another within the CIJ mixer by syringe injection. This process was repeated once more using 15 mL of water and the obtained 30 mL of suspension as the two impingement solutions. After the two-step hydration, 45 mL of suspension was collected, followed by dialysis in water (3 days) to remove DMSO. As prepared, the NPs (suspended in water) were stored at room temperature before using.

### Cryo-scanning transmission electron microscopy (cryo-STEM).

Lacey carbon grids (200 mesh, Electron Microscopy Sciences, Inc.) were glow-discharged for 20 s in a Pelco easiGlow glow-discharger (15 mA, 0.24 mBar). Each sample (4 uL) was pipetted onto a grid and plunge-frozen into liquid ethane using an FEI Vitrobot Mark IV cryo plunge freezing robot with a blotting time of 5 or 5.5 seconds and blotting pressure of 1. Frozen grids were stored in liquid nitrogen and then loaded into a Gatan 626.5 cryo transfer holder cooled down to −180 °C prior to image within a Hitachi HD2300 STEM at 200kV. Frozen samples were previewed with the phase contrast TE and HAADF detectors to verify sample preparation. With the sample still in the microscope, the cryo holder was slowly warmed up to −95 °C over the course of 30 minutes to sublime away ice within the vacuum. Imaging in the microscope continued using an SE detector in the STEM to image the surface of our particles. Image data was collected with Gatan Digital Micrograph and a Digiscan system. Any further image processing conducted on the aligned frames was completed in ImageJ.

### All atom explicit solvent molecular dynamics simulations.

All simulations were performed with GROMACS 2021.5^24^, an MD package mainly designed for simulations of large biomolecules. The CHARMM 36m force field^25^ was used for the all-atom simulations, which has been validated in our last work^8^. The recommended CHARMM TIP3P water model was applied with the structures constrained using the SETTLE algorithm^26^. All visual analyses were performed with VMD^27^. In all the simulations, the degree of polymerization of 20 was employed for the polymer chains, the same as that PPSU_20_ in the experiments.

### Preparation of protein@NP complex via protein adsorption.

Except for antibodies, excess protein was used to achieve full cover on NP surfaces. (i) NPs (5 mg/mL) were suspended in protein solution (10 mg/mL in PBS) for 5 min; (ii) they were centrifuged, and the NP pellet was collected; (iii) the pellet was resuspended in PBS, followed by centrifugation. Step (iii) was repeated a total of three times to remove unbound proteins. The final protein@NP complex was suspended in PBS.

### Trypsin proteolysis fluorescence-based kinetic assay.

2 ug/ml of trypsin (free protease or nanoparticulate form) was incubated with 1 mg/mL of quenched FITC-BSA substrate at 37 °C. Trypsin cleaves yields FITC-labeled peptides derived from the full-length protein, which releases the fluorophore from a quenched state and results in an increase in fluorescence. Proteolysis was therefore monitored by measuring the increase in fluorescence over a 1.5 h period. (1 measurement per minute). Measurements were obtained in three replicates per condition.

### SAXS measurements.

SAXS measurements were performed at the DuPont-Northwestern-Dow Collaborative Access Team (DND-CAT) beamline at Argonne National Laboratory’s Advanced Photon Source (Argonne, IL, USA) with 10 keV (wavelength λ = 1.24 Å) collimated X-rays. All the samples (5 mg/mL) were analyzed in the q-range (0.001–0.5 Å^−1^), with a sample-to-detector distance of approximately 8.5 m and an exposure time of 5 s. The beamline was calibrated using silver behenate and gold coated silicon grating with 7,200 lines/mm The momentum transfer vector q is defined as q = 4πsinθλ^−1^, where 2θ is the scattering angle. Data reduction and buffer subtraction were performed using IRENA 2.71 package within IGOR PRO 9 software (Wavemetrics). Model fitting was completed using SasView 5.0.5 software package wherecore-shell sphere model was utilized to fit and analyze the data.

### MALDI-TOF mass spectrometry detection of trypsin.

Trypsin (5 wt.%) was encapsulated or adsorbed by PPSU NPs. As prepared, the samples were stored for 48 h at 4 °C to permit leakage of weakly associated trypsin (if present). The samples were pelleted by centrifugation at 20,000 × g for 10 min at room temperature. The supernatants were collected and were mixed 1:1 with sinapinic acid matrix prepared in ultrapure water with 0.2% trifluoroacetic acid. Trypsin prepared at 2 ug/mL was used as a positive control for detecting full length protein. The supernatant collected from PPSU NPs prepared in the absence of protein was used as a negative control. These mixtures were spotted on a stainless steel MALDI disc (Bruker), and full-length trypsin protein (~23 kDa) was detected by MALDI-TOF mass spectrometry. Positive ion mass spectra were collected on a Bruker rapifleX MALDI Tissuetyper operating in linear TOF mode. Spectra were obtained in triplicate.

### Immunogold labelling.

Samples were prepared by mixing 90 μL of PPSU NPs (1 mg/mL in water), 10 μL of anti-CD4 antibodies (0.5 or 0 mg/mL in PBS), 10 μL of 10×PBS, and 100 μL of BSA (1 mg/mL in PBS). After incubation for 5 min at 4 °C, the samples were centrifuged, and the collected pellets were resuspended in PBS. This step was repeated a total of three times to remove unbound protein molecules. 20 μL of secondary gold-coupled antibodies (with 1 wt.% of BSA in PBS) was added into each of the samples and the mixtures were dialyzed in water to remove salts. Negatively stained grids were prepared using 0.5 mg/mL of NPs in water, then visualized on a JOEL 1400 TEM operating at 120 kV.

### Cellular uptake studies.

Rhodamine B-encapsulated NPs were mixed with 5 wt.% of anti-CD3 antibodies (or Isotype control, 10 ug/mL) in PBS for 5 min, then suspended in PBS with 10mg/mL of BSA. Unbound proteins were removed by washing with PBS. The antibody-adsorbed NPs (25 μg/mL) were incubated with Jurkat T cells for 30 min at 37 °C. Cells were analyzed using a BD Fortessa flow cytometer. The cellular uptake was measured as % of NP positive cells and median fluorescence intensity (MFT).

### Preparation of formulations for *in vitro* and *in vivo* validation experiments.

Stocks of antibody-adsorbed NPs were prepared by concurrently incubating PPSU NPs with set concentrations of anti-FcεRIα (0.1 wt.% for *in vitro* or 0.2 wt.% for *in vivo*) and variable amounts of anti-Siglec-6 to create NPs for co-engagement. Anti-Siglec-6 concentrations for *in vitro* experiments were determined by loading different weight percentages of the total PPSU mass present in each sample (0.01, 0.1, and 1 wt.%). This translated to final well concentrations of 8 ng/mL, 80 ng/mL, and 800 ng/mL, respectively. In mouse experiments, we utilized 2.5 wt.% anti-Siglec-6 (5 μg per injection). When preparing PPSU NPs with multiple antibodies, the protein was added to a microtube in the desired ratios and concentrations before addition of PPSU NPs. After incubation of antibodies for 5 min, NPs were incubated with excess BSA before final washes to remove unbound protein.

### Mast cell isolation and cell culture.

Skin-derived primary mast cells (SkMCs) were isolated from 100 cm^2^ human skin samples received from the Midwestern Division of the Cooperative Human Tissue Network. Multiple donors were used across our experiments to ensure consistency despite patient variability. We utilized a protocol developed by Caslin et al.^28^; in brief, we cut skin into <1 cm^2^ pieces and then subjected tissue to a digestive enzyme wash at 37 °C for 1 hour, completing 3 rounds of washes. After this, cells were pelleted under centrifugation and further purified by collecting a buffy coat over percoll. Cells were then purified (>95%) via passive media selection in culture for 8 weeks before use in experiments.

### Scanning electron microscopy (SEM).

The medium was removed after cell incubation, and glutaraldehyde solution (2.5%) was added for fixation overnight at 4 °C. The cells were then rinsed three times with 0.1M of sodium cacodylate buffer and once with water (5 min) and incubated in osmium tetroxide (2%) for 1 hour. After staining, the cells were rinsed another three times with water (10 min per time). The cells were dehydrated with a series of ethanol gradients (30, 50, 70, 85, and 95%) for 10 min at each step. The dehydrated cells were rinsed with 100% ethanol two times (10 min per time) and was completely dried using Samdri^®^−795 - tousimis Critical Point Dryers. After drying, the cells were placed on a carbon tape attached to an aluminum stud and coated with SPF Osmium Coater (10 nm thickness) for SEM analysis. The morphology was then observed by a Hitachi SU8030 scanning electron microscope.

### Analysis of degranulation markers with flow cytometry.

In preparation for the experiments, we used dose curves to optimize the concentration of anti-FcεRIα required to induce clear expression of degranulation markers on SkMCs above baseline levels (80 ng/mL). Additionally, we incubated the cells with 10 ng/mL of polyclonal human IgE (Abcam) O/N to stimulate FcεRI expression and prime cells for activation. For the experiment, SkMCs were resuspended in ice cold Tyrode’s buffer at 300K cells/sample and given a variety of experimental formulations containing PPSU NPs, free or antibody-adsorbed NPs, or combinations of each. Mouse IgG2a isotype antibody (BioLegend clone MG2a-53) was used as a control comparison for anti-Siglec-6. Cells were placed in a 37 °C incubator for 20 minutes before being placed immediately back on ice to halt ongoing degranulation. Cells were then washed with PBS and suspended with live/dead fixable blue dead cell stain for 30 minutes at 4 °C. Subsequently, cells were washed with FACS buffer (PBS with 3–4% FBS) and resuspended with blocking buffer for 10 minutes and then directly added master mix of antibody labels to incubate for 45 minutes at 4 °C. At last, cells were washed 2 times with FACS buffer or fixed with 4% PFA and then washed, depending on equipment availability. All flow sample data were collected on a BD FACSymphony and simultaneously compensated using the equipped X software. Antibodies: BV421 labeled anti-CD117 (KIT), clone 104D2 from BioLegend. PE labeled anti-Siglec-6, clone 767329 from R&D Systems, PE/Cy7 labeled anti-CD63, clone H5C6, from BioLegend, APC labeled anti-CD107a (lamp1), clone H4A3, from BioLegend, and APC/Cy7 labeled anti-FcεRIα, clone AER-37, from BioLegend. Samples were analyzed on FlowJo v10. All samples were gated for singlets, then live/dead and normal KIT expression before analyzing other markers. Additionally, all samples were gated to a minimum of 15,000 KIT+ cells.

### Cytokine expression analysis via enzyme-linked immunosorbent assay (ELISA).

Human TNF-α (cat. #QTA00C) ELISA kits were obtained from R&D systems. 100K SkMCs cells/well (n = 4 per test group) were incubated with formulations for 3 hours. The supernatant was then collected and stored overnight at −20 °C before being used in kits as prescribed in their protocols. The same conditions were utilized for our other *in vitro* experiments. Here, we dosed cells with 0.1 wt.% anti-Siglec-6 (80 ng/mL) on PPSU NPs, with a final well concentration of 80 μg/mL of PPSU NPs (8 μg per sample). Just as with the flow cytometry samples, anti-FcεRIα was adsorbed onto PPSU NPs or dosed separately at 80 ng/mL.

### Humanized mouse engraftment and anaphylaxis model.

We utilized a previously established and characterized model to test the inhibitory effects of our formulations *in vivo*^23^. Four-week-old NSG-SGM3 mice were retro-orbitally injected with hCD34+ stem cells. After 16 weeks, mice were checked for engraftment using flow cytometry. We used FITC labeled anti-hCD45 clone 2D1 (BioLegend) and APC/Cy7 labeled anti-mCD45 clone 103116 (BioLegend) to compare human and mouse cell populations. Successfully engrafted mice (> 5% hCD45+) were then randomized with respect to the percentage of engraftment and retro-orbitally injected with test formulations. Nanoparticulate and free anti-FcεRIα (1 μg/mL) was used as a positive control in comparison to mice given the nanomedicine and the other test formulations. Visual clinical scoring and a rectal temperature probe were used to track anaphylactic reaction severity for 1-hour post-injection, with data collected at 10-minute intervals. Clinical scores were determined as follows: 1: scratching, 2: piloerection/facial edema, 3: labored breathing, 4: comatose/unresponsive 5: death. Mice were then sacrificed and not reused.

## Figures and Tables

**Fig. 1:**
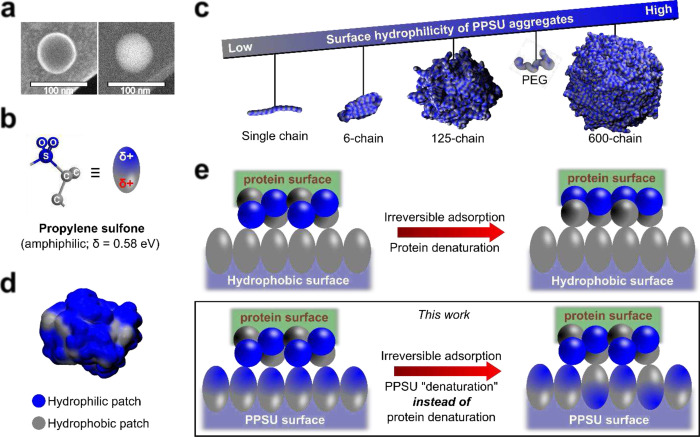
Protein surfaces induce site-to-site dipole rotation of interfacial propylene sulfone. **(a)** Simultaneously recorded cryo-STEM and cryo-SEM images showing a typical PPSU hollow nanoparticle and its surface, respectively. **(b)** Schematic illustration of the dipole moment of amphiphilic propylene sulfone, the repeating unit of PPSU homopolymer (blue indicates hydrophilic and grey indicates hydrophobic for all the schemes in the work, unless stated otherwise). **(c)** Atomistic simulations demonstrating enhanced surface hydrophilicity as the aggregation of PPSU chains become disordered. PEG is included for comparison. **(d)** As seen in trypsin using glycine as the reference, the surfaces of water-soluble proteins are heterogeneous, with characteristic patch size distributions based on hydrophobicity. **(e)** Protein inducing the formation of locally heterogeneous PPSU surfaces that mimic protein surfaces. Hydrophobic interactions at the interface between this PPSU surface and the adsorbed protein can be weak enough not to outcompete the forces governing protein folding despite irreversible adsorption. Fast spreading of protein on a hydrophobic surface is included for comparison^2^.

**Fig. 2:**
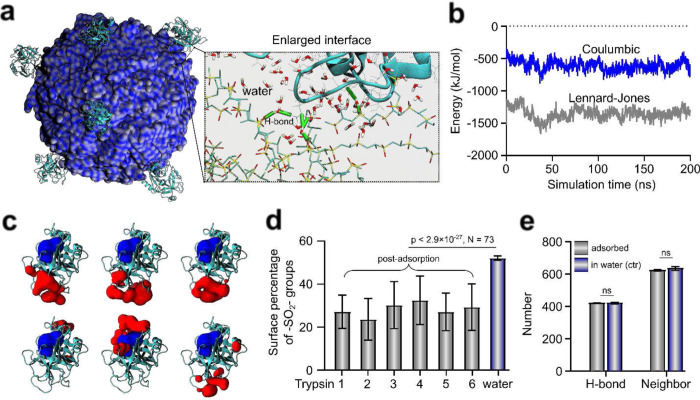
Atomistic explicit solvent simulations confirm the interfacial hydrophilic-hydrophobic transition within PPSU surfaces upon trypsin adsorption. **(a)** Simulation snapshot of equilibrated 600-chain NP adsorbed with all the 6 trypsin molecules (in cyan). The protein-NP interfaces are lubricated by water molecules, leading to the preserved structure of trypsin. The green cylinders refer to the water bridges connecting PPSU chains and trypsin via H-bonds. **(b)** The Lennard-Jones trypsin-NP interactions dominated over the trypsin-NP Coulombic interactions, supporting the hydrophobicity-driven feature of trypsin adsorption. **(c)** Orientations of the 6 trypsin molecules are non-specific upon adsorption. The adsorption sites and active sites on trypsin are colored in red and blue, respectively. **(d)** Percentage of sulfone groups at the trypsin-NP contact region revealing enhanced NP surface hydrophobicity after trypsin adsorption. **(e)** No significant (ns) differences in trypsin hydration were detected between the adsorbed trypsin and unbound trypsin. The numbers of trypsin-water H-bonds and of water neighbors of trypsin were calculated. Error bars represent standard deviations. Statistical significance is determined by Tukey-post hoc test.

**Fig. 3:**
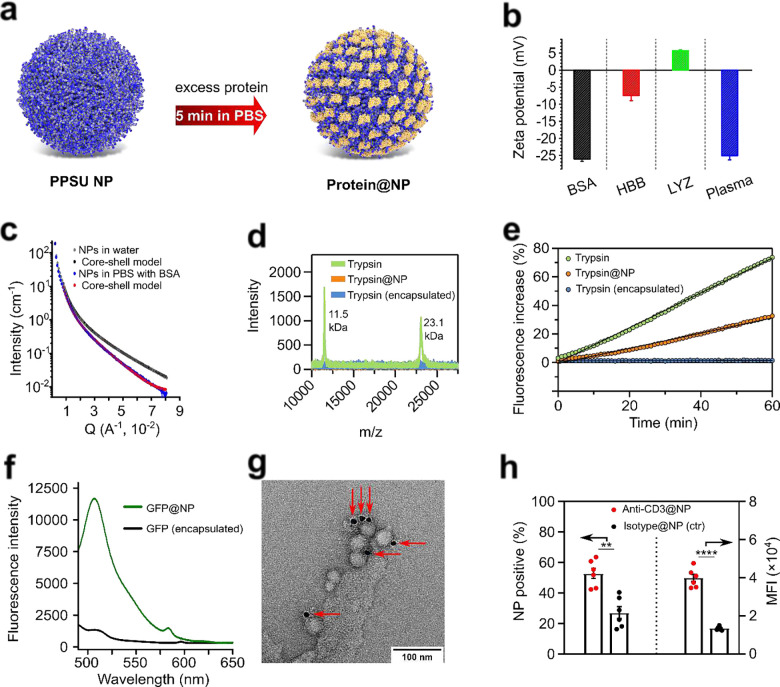
PPSU NPs preserve protein function within stable adlayers. **(a)** Schematic illustration of the rapid and facile process of coating PPSU NPs with protein adlayers. After incubation, unbound proteins are removed by thorough washing. **(b)** The zeta potential of protein-coated NPs is dependent on the adsorbed proteins. **(c)** SAXS data supporting the increase of shell thicknesses from 5.3 nm to 7.1 nm after BSA adsorption. **(d)** Stability of trypsin@NP. Desorption of trypsin from the nanozymes is undetectable during storage at 4 °C for 48 h by MALDI-TOF mass spectrometry. **(e)** Kinetic assay demonstrating bioactivity of adsorbed trypsin. Trypsin is encapsulated within the NPs for comparison. **(f)** Fluorescence of GFP was detectable for GFP@NP but not for GFP-encapsulated NPs. **(g)** Immunogold labelling showing the binding of pre-adsorbed anti-CD4 antibodies to secondary antibodies. 10 nm colloidal gold–secondary antibody, indicated by the arrows. **(h)** Targeting of irreversibly adsorbed anti-CD3 towards Jurkat T cells is confirmed by flow cytometry as assessed by percentage of NP positive cells and median fluorescence intensity (MFI). Statistical significance is determined by Tukey-post hoc test: * p < 0.005, ** p < 0.001, *** p < 0.0005, **** p < 0.0001.

**Fig. 4:**
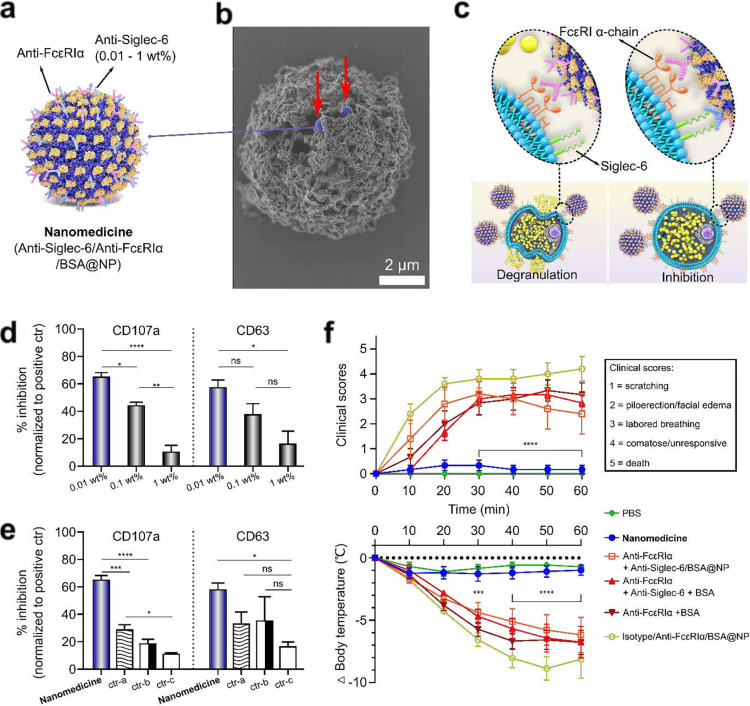
Optimization of multi-antibody coatings allows mast cell targeting for anaphylaxis nanotherapy. **(a)** PPSU-based nanomedicines consisting of co-adsorbed anti-Siglec-6 and anti-FcεRIα with controlled surface density (as wt% of PPSU) of anti-Siglec-6. **(b)** Imaging the nanomedicines (blue, indicated by red arrows) on the surface of a mast cell (grey) by SEM. **(c)** Activation of mast cells (left) is achieved via cross-linking of FcεRIα by anti-FcεRIα/BSA@NP, whereas the nanomedicine inhibits mast cell degranulation (right) via co-localized engagement. **(d)** Optimizing nanomedicine formulation via adjusting the surface density of anti-Siglec-6. *In vitro* results showing that lower anti-Siglec-6 density is more effective in suppressing CD107a and CD63 expression. **(e)** *In vitro* results demonstrating the importance of binding Siglec-6 in close proximity and time with engagement of FcεRI in suppressing CD107a and CD63 expression by mast cells. The optimized formulation (0.01 wt% of anti-Siglec-6) is used for the nanomedicine; ctr-a: Anti-Siglec-6/BSA@NP + Anti-FcεRIα; ctr-b: Anti-Siglec-6 + Anti-FcεRIα + BSA; ctr-c: BSA@NP + Anti-FcεRIα. (d-e) Inhibition is normalized from mast cells receiving only anti-FcεRIα and expressing a positive population mean of 52.7 ± 5% for CD63 and 71.2 ± 8% for CD107a (n = 3). **(f)** The optimized nanomedicine (0.01 wt% of anti-Siglec-6) succeeds in administering allergen immunotherapy without triggering anaphylaxis in a humanized mouse model. Results were from 2 combined datasets (total n = 6). Statistical significance is determined by Tukey-post hoc test: * p < 0.05, ** p < 0.01, *** p < 0.005, **** p < 0.001.
